# Analysis of the effects of individualized nutrition and physical activities on body composition during the treatment of breast cancer patients

**DOI:** 10.2478/raon-2026-0046

**Published:** 2026-09-07

**Authors:** Rok Blaz, Masa Kovac, Nada Rotovnik Kozjek

**Affiliations:** 1 University of Ljubljana, Medical Faculty, Ljubljana, Slovenia; 2 Institute of Oncology Ljubljana, Department for Clinical Nutrition, Ljubljana, Slovenia

**Keywords:** individualized nutrition, physical activity, body composition, breast cancer, bioelectrical impedance analysis, GLIM criteria

## Abstract

**Background:**

Malnutrition and loss of skeletal muscle mass are common in breast cancer patients and represent negative prognostic factors. Combined nutrition and exercise interventions may help preserve body composition during treatment. The aim of this study was to evaluate the effects of a targeted nutritional and exercise intervention on body composition, assessed by Bioelectrical Impedance Analysis (BIA), in breast cancer patients undergoing treatment, stratified by baseline nutritional status according to the Global Leadership Initiative on Malnutrition (GLIM) criteria.

**Patients and methods:**

In this retrospective analysis of the OREH pilot study, 287 patients were included in the intervention group and 194 in the control group. All patients underwent BIA measurements at baseline, 6 months, and 12 months. Malnourished patients (GLIM-positive) in both groups received individualized nutritional counseling as standard care. GLIM-negative patients in the intervention group received preventive nutritional counseling via online lectures. All intervention group patients participated in supervised group exercise. Differences in BIA parameters (fat-free mass index, phase angle, fat mass, total body water, and body mass index) between the 3rd and 1st measurements were compared using Welch’s t-test, with p-values adjusted by the Bonferroni-Holm method.

**Results:**

No statistically significant differences in body composition parameters were observed between the intervention and control groups over the 12-month follow-up period. The adjusted p-value for fat-free mass index (FFMI) was 0.2 in GLIM-positive patients and 1.0 in GLIM-negative patients. A trend toward greater FFMI increase was observed in GLIM-positive patients in the intervention group compared to controls (mean difference 0.30; 95% CI 0.05–0.54; unadjusted p = 0.017), but this did not remain significant after correction for multiple comparisons.

**Conclusions:**

The combined nutritional and exercise intervention did not significantly improve BIA-derived body composition parameters. However, the trend toward FFMI improvement in GLIM-positive patients receiving individualized nutritional counseling suggests that personalized approaches may be more effective than group-based interventions. Future studies should incorporate individualized nutrition and resistance training programs to optimize body composition outcomes in breast cancer patients.

## Introduction

Breast cancer is one of the most common cancers among women and represents a major public health challenge worldwide. The World Health Organization estimates that in 2022, approximately 2.3 million women were diagnosed with breast cancer and 670,000 died from the disease, making it the most common cancer in women in 157 of 185 countries.^1,2^ In Slovenia, breast cancer is the most frequently diagnosed cancer in women, with 1,660 new cases, 470 deaths, and 7,128 five-year prevalent cases reported in 2022.^1,3^ The national screening program DORA has achieved nationwide coverage with participation rates consistently above 70%, and registry-based evaluations have demonstrated that women undergoing organized screening have lower cancer stage at diagnosis and significantly better five-year net survival.^4,5^

Malnutrition is a prevalent and clinically consequential complication in cancer patients undergoing active treatment. It is defined as a state of energy and protein deficiency leading to unfavorable changes in body composition and impaired physiological function.^6^ A more severe manifestation is cancer cachexia – a multifactorial syndrome characterized by ongoing loss of skeletal muscle mass, with or without fat mass depletion, that cannot be fully reversed by conventional nutritional support.^6^ Cachexia develops through complex pathophysiological mechanisms, including systemic inflammation driven by pro-inflammatory cytokines, accelerated proteolysis (the enzymatic breakdown of muscle proteins), and anabolic resistance (the diminished capacity of skeletal muscle to respond to anabolic stimuli such as protein ingestion or exercise).^6,7^ These processes are exacerbated by inflammatory factors released during cancer growth and are further intensified by chemotherapy and surgical treatment.^8^ Additional contributing factors include comorbidities, treatment-related side effects (anemia, loss of appetite, nausea, vomiting, diarrhea, oral ulcers, taste changes, and dysphagia), and cessation of physical activity.^9^ Collectively, these factors lead to quantitative and qualitative muscle loss, increased frailty, higher mortality, and decreased quality of life.^10-13^

Body composition has emerged as a critical prognostic factor in cancer patients.^14^ Fat-free mass (FFM) represents metabolically active tissues, including muscles, bones, and visceral organs, and its loss is associated with higher mortality, poor clinical outcomes, and worse well-being.^14^ Importantly, body mass index (BMI) alone fails to capture compositional changes, as patients may develop sarcopenic obesity. Sarcopenic obesity represents the simultaneous loss of muscle mass and gain of fat mass during treatment, characterized by reduced fat-free mass index (FFMI 15 kg/m^2^ for women, 17 kg/m^2^ for men) with normal or increased body weight.^15^ Bioelectrical Impedance Analysis (BIA) is a simple, non-invasive method for assessing body composition that can be used at the bedside.^14^ BIA measures tissue impedance when an electric current passes through the body: at lower frequencies, the current bypasses cell membranes, providing an estimate of extracellular water, while at higher frequencies it penetrates cell membranes, allowing assessment of total body water (TBW).^14^ From these measurements, FFM, fat mass (FM), and phase angle can be calculated. Phase angle, obtained directly from BIA measurements, reflects cell membrane integrity and the ratio of intracellular to extracellular water. It is a reliable indicator of body cell mass, is strongly associated with muscle strength, and has high prognostic value in cancer patients.^16^ However, since BIA is an indirect method, its reliability depends on certain conditions, including homogeneous tissue composition, stable fluid balance, and BMI between 16 and 34 kg/m^2^, which may not always be met in hospitalized or chronically ill patients.^16^

The role of nutrition and exercise interventions in mitigating treatment-related body composition changes has gained increasing attention. Personalized nutrition is an important component of managing malnutrition in cancer patients, as standard dietary recommendations are not appropriate for oncology patients whose nutritional requirements differ from those of healthy adults.^10^ A daily protein intake of at least 1 g/kg body weight from high-quality sources is recommended.^10^ However, nutritional interventions alone are insufficient to fully prevent loss of body mass. Planned physical activity improves cardiorespiratory, neuromuscular, and immune system function, and has positive psychological effects.^12^ Exercise increases gene expression of myogenic factors, activates mitochondrial biogenesis, reduces systemic inflammation and oxidative stress, counteracts cancer-induced anabolic suppression, and promotes protein synthesis.^13^ At least 150 minutes of moderate-intensity or 75 minutes of vigorous-intensity exercise per week is recommended for breast cancer patients, with resistance exercises advised at least two to three times per week to maintain or increase muscle function and mass.^9^

The assessment of nutritional status in cancer patients begins with screening for nutritional risk using validated tools such as the Nutritional Risk Screening 2002 (NRS 2002).^17^ If a patient is found to be at nutritional risk, the diagnosis of malnutrition can be established using the Global Leadership Initiative on Malnutrition (GLIM) criteria.^18^ The GLIM criteria have been validated in clinical settings, demonstrating their ability to predict adverse clinical outcomes and response to nutritional treatment.^19^

Despite growing evidence supporting nutrition and exercise interventions in oncology, important knowledge gaps remain. Few studies have evaluated individualized, combined nutrition and exercise interventions targeting body composition specifically in breast cancer patients undergoing active treatment. Furthermore, the interaction between baseline nutritional status defined by GLIM criteria and intervention response remains poorly characterized. Preliminary data from a pilot study on individualized comprehensive rehabilitation in breast cancer patients in Slovenia suggested potential benefits, but definitive evidence is lacking.^20^

The aim of this study was to evaluate the effects of a targeted online nutrition sessions and supervised exercise intervention on body composition parameters assessed by BIA in breast cancer patients undergoing treatment, stratified by baseline nutritional status according to GLIM criteria.

## Patients and methods

### Study design and participants

The study was conducted as part of the Pilot Study on Individualized Comprehensive Rehabilitation of Breast Cancer Patients (OREH Study) at the Institute of Oncology Ljubljana (OIL), between 2019 and 2022.^20^ A total of 600 patients were enrolled, with 301 assigned to the control group and 299 to the intervention group. Due to incomplete BIA measurements, the final sample consisted of 287 patients in the intervention group and 194 patients in the control group.

The intervention group included women aged 25 to 65 years diagnosed with breast cancer, treated at OIL, with permanent residence within the regional units of the Health Insurance Institute of Slovenia (ZZZS), Ljubljana or Kranj.^20^ The control group comprised women with breast cancer aged 25 to 65 years, with permanent residence within the ZZZS regional units of Nova Gorica, Koper, Novo Mesto, Krško, Ljubljana, or Kranj.^20^

### Intervention

A permanent reference point was established at OIL by a coordination nurse, who served as the central contact person to provide guidance and support during treatment. The comprehensive rehabilitation program included online workshops and off-site services delivered in collaboration with the University Rehabilitation Institute Soča, the Institute for Medical Rehabilitation at the University Medical Center Ljubljana, and Health Promotion Centers within community health centers.^20^ Approval for the study was granted by the National Medical Ethics Committee of the Republic of Slovenia (reference number: 0120-264/2024-2711-3).

Online activities for patients in the intervention group included:

1. Clinical nutrition lectures (five series, each consisting of three one-hour sessions) delivered by clinical dietitians from the Clinical Nutrition Unit of OIL (average attendance per session: 31 participants).
2. Supervised group exercise sessions, conducted twice weekly for 30 minutes under the supervision of kinesiologists from the Faculty of Sport (average attendance: 25 participants).
3. Yoga sessions, conducted once weekly under the supervision of a physiotherapist (average attendance: 35 participants).

### Nutritional assessment and management

All patients in both groups were screened for nutritional risk using the NRS 2002 tool.^17^ Patients identified as at nutritional risk underwent diagnostic assessment using the GLIM criteria, which require at least one phenotypic criterion (unintentional weight loss, low BMI, or reduced FFMI) and at least one etiologic criterion (reduced dietary intake or disease burden/inflammation) for the diagnosis of malnutrition.^18,21^ All patients diagnosed with malnutrition (GLIM-positive) in both groups were referred for individualized nutritional management, which represents the standard clinical practice at OIL.^21^

### Body composition measurements

In both groups, body composition measurements were performed using BIA at baseline (measurement 1), at 6 months (measurement 2), and at 12 months (measurement 3). Measurements were obtained with a Bodystat Quadscan 4000 device. The following parameters were monitored: BMI (kg/m^2^), FFMI (kg/m^2^), phase angle (degrees), TBW (%), and FM (%).

### Statistical analysis

Continuous variables were summarized as means and standard deviations, and categorical variables as frequencies and percentages. Baseline demographic and clinical characteristics were compared between the intervention and control groups using the chi-square test or Fisher’s exact test for categorical variables, as appropriate, and Welch’s two-sample t-test for continuous variables. The primary outcome was the change in body composition parameters between the third and first measurement. The analyzed BIA parameters were fat-free mass index, body mass index, total body water, fat mass, and phase angle. Positive values indicated an increase between baseline and the third measurement. Changes in BIA parameters were compared between the intervention and control groups using Welch’s two-sample t-test, separately for GLIM-negative and GLIM-positive patients. To account for multiple comparisons across BIA parameters and GLIM subgroups, p-values were adjusted using the Bonferroni-Holm method. All tests were two-sided, and adjusted p-values below 0.05 were considered statistically significant. Missing data was not imputed because the proportion of missing observations was low (3.4% across all outcome variables and measurement points). Missing outcome data were handled using pairwise deletion. Statistical analyses were performed using R software.

## Results

### Baseline characteristics

The study included 481 female patients aged 25 to 65 years (287 intervention, 194 control). The mean age at baseline was 51.3 ± 7.1 years in the intervention group and 51.2 ± 7.3 years in the control group, with no statistically significant difference (p = 0.909). Baseline demographic and clinical characteristics are presented in Table 1.

**TABLE 1.** Baseline demographic and clinical characteristics of participants

		Intervention	Control	p
N		287	194	
**Cancer stage [%]**				0.398
	0	5 (1.7)	4 (2.1)	
	1	121 (42.2)	86 (44.3)	
	2	114 (39.7)	64 (33.0)	
	3	30 (10.5)	30 (15.5)	
	4	17 (5.9)	10 (5.2)	
**Living environment [%]**				0.001
	urban	157 (54.7)	78 (40.2)	
	suburban	48 (16.7)	30 (15.5)	
	rural	82 (28.6)	86 (44.3)	
**Social status [%]**				0.836
	upper class	4 (1.4)	3 (1.5)	
	upper middle class	44 (15.3)	25 (12.9)	
	middle class	205 (71.4)	141 (72.7)	
	working class	33 (11.5)	23 (11.9)	
	lower class	1 (0.3)	1 (0.5)	
	no data	0 (0.0)	1 (0.5)	
**Age at baseline (years) ± SD**		51.28 +/- 9.11	51.19 +/- 8.76	0.909
**Nutritional Risk Screening (NRS) [%]**		122 (42.5)	90 (46.4)	0.455
**NRS – points [%]**				
	0	165 (57.5)	104 (53.6)	
	1	90 (31.4)	66 (34.0)	
	2	13 (4.5)	12 (6.2)	
	3	7 (2.4)	4 (2.1)	
	4	1 (0.3)	0 (0.0)	
	no data	11 (3.8)	8 (4.1)	
**GLIM -positive [%]**		60 (20.9)	52 (26.8)	0.164
**Obesity = 1 [%] [BMI ≥ 30 kg/m^2^]**		61 (21.3)	41 (21.1)	1.000

1 BMI = body mass index; GLIM = Global Leadership Initiative on Malnutrition; NRS 2002 = Nutritional Risk Screening 2002; SD = standard deviation; WHO = World Health Organization

Baseline characteristics were largely similar between groups. There were no statistically significant differences in cancer stage (p = 0.398), social status (p = 0.836), nutritional risk by NRS 2002 (p = 0.455), GLIM-defined malnutrition (20.9% *vs*. 26.8%; p = 0.164), or obesity prevalence (21.3% *vs*. 21.1%; p = 1.000). However, the living environment differed significantly between groups, with a higher proportion of urban participants in the intervention group (54.7%) compared to the control group (40.2%), and a higher proportion of rural participants in the control group (44.3% *vs*. 28.6%; p = 0.001).

Among patients screened with NRS 2002, 42.5% of the intervention group and 46.4% of the control group were identified as at nutritional risk (p = 0.455). The distribution of NRS scores was similar between groups, with the majority scoring 0 (no risk) or 1 point

### Body composition measurements

Descriptive statistics for BIA parameters at each measurement time point, stratified by GLIM status and group allocation, are presented in Table 2. Measurement time points correspond to baseline (measurement 1), 6 months (measurement 2), and 12 months (measurement 3). In GLIM-negative patients, FFMI remained relatively stable across all three measurements in both groups (intervention: 17.1, 17.2, 17.0 kg/m^2^; control: 17.2, 17.2, 17.1 kg/m^2^). Phase angle decreased from baseline to 6 months in both groups (intervention: 5.4 to 4.9; control: 5.5 to 5.0), with partial recovery at 12 months (intervention: 5.0; control: 5.2). Fat mass percentage and TBW remained largely unchanged.

**TABLE 2.** Descriptive statistics (mean ± SD) for Bioelectrical Impedance Analysis (BIA) parameters at each measurement time point, stratified by GLIM status

		GLIM negative		GLIM positive	
Parameter	Meas.	Intervention	Control	Intervention	Control
Phase angle (°)	1	5.4 +/-0.7	5.5 +/-0.6	5.2 +/-1.3	5.2 +/-0.6
	2	4.9 +/-0.6	5 +/-0.6	4.8 +/-0.6	4.7 +/-0.7
	3	5 +/-0.7	5.2 +/-0.7	4.9 +/-0.7	4.9 +/-0.6
FFMI (kg/m^2^)	1	17.1 +/-1.5	17.2 +/-1.3	15 +/-1.7	15 +/-1.6
	2	17.2 +/-1.7	17.2 +/-1.5	15.5 +/-1.8	15.4 +/-1.8
	3	17 +/-1.6	17.1 +/-1.4	15.4 +/-1.6	15.1 +/-1.5
Fat mass (%)	1	36.5 +/-7.5	36.6 +/-7	30.3 +/-7.4	30.4 +/-8.5
	2	36.3 +/-7.1	36.6 +/-7	31.4 +/-6.7	30.3 +/-6.3
	3	37 +/-6.8	37.1 +/-7.1	32.4 +/-8.4	31.3 +/-6.5
TBW (%)	1	47.6 +/-5	47.6 +/-4.9	53.6 +/-5.7	54 +/-6.1
	2	48 +/-5	47.8 +/-4.8	52.8 +/-4.9	53.6 +/-5.4
	3	47.5 +/-4.7	47.4 +/-4.9	51.5 +/-5.3	53.2 +/-5.8

1 FFMI = fat-free mass index; GLIM = Global Leadership Initiative on Malnutrition; TBW = total body water Meas. = Measurement; Measurement 1 = baseline; Measurement 2 = 6 months; Measurement 3 = 12 months

**FIGURE 1.**
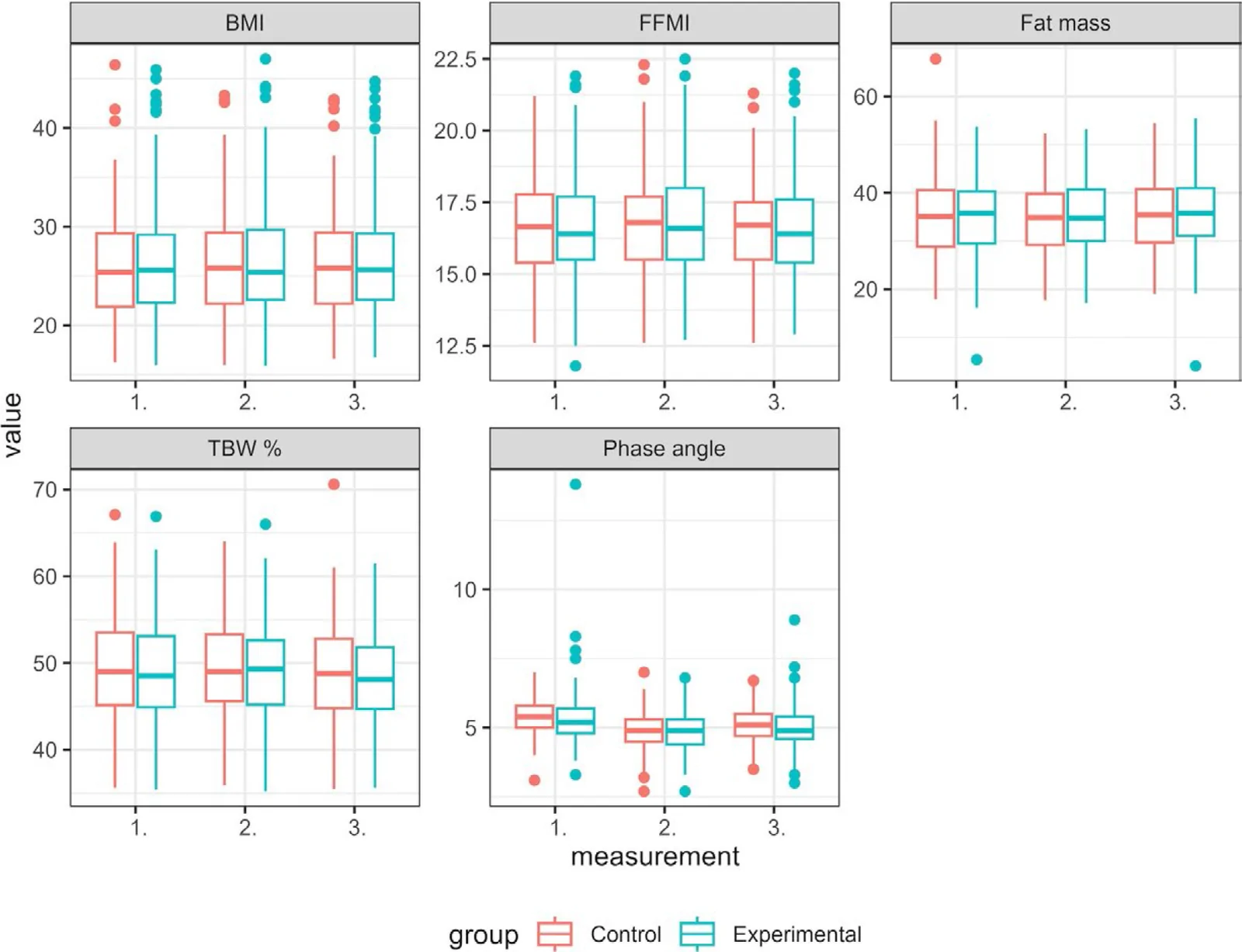
Distribution of Bioelectrical Impedance Analysis (BIA) measurements. The median and the first and third quartiles of each measurement are shown. BMI = body mass index; FFMI = fat-free mass index; TBW = total body water Measurement 1 = baseline; Measurement 2 = 6 months; Measurement 3 = 12 months

In GLIM-positive patients, FFMI increased from baseline to 6 months in both groups (intervention: 15.0 to 15.5 kg/m^2^; control: 15.0 to 15.4 kg/m^2^), with the intervention group maintaining a slightly higher value at 12 months (15.4 *vs*. 15.1 kg/m^2^). Phase angle decreased similarly in both groups from baseline to 6 months (intervention: 5.2 to 4.8; control: 5.2 to 4.7), with partial recovery at 12 months (both groups: 4.9). Fat mass percentage increased progressively in both groups, with a greater increase in the intervention group (30.3 to 32.4%) compared to controls (30.4 to 31.3%). TBW decreased in both groups, with a more pronounced decrease in the intervention group (53.6 to 51.5%) compared to controls (54.0 to 53.2%). The distribution of BIA measurements at each time point is shown in Figure 1.

### Comparison of body composition changes between groups

**FIGURE 2.**
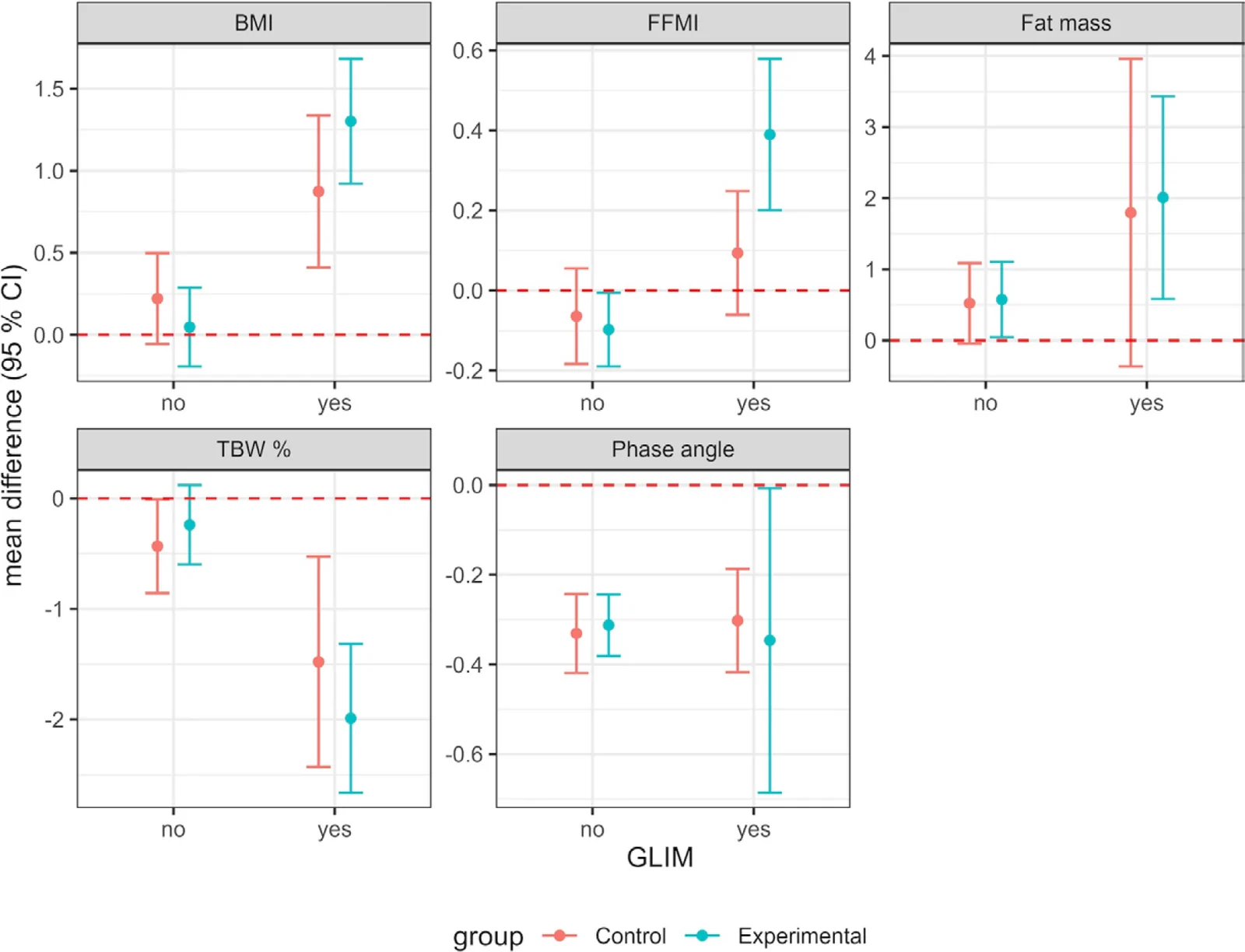
Mean difference between the 3rd and 1st measurement for each parameter with 95% confidence interval, shown for GLIMpositive and GLIM-negative patients in the control and intervention group. BMI = body mass index; FFMI = fat-free mass index; GLIM = Global Leadership Initiative on Malnutrition; TBW = total body water

To assess the effect of the intervention, the difference between the 3rd (12 months) and 1st (baseline) measurements was calculated for each parameter. The mean difference and 95% confidence interval are presented in Figure 2, stratified by GLIM status and group allocation.

Differences between groups were tested using Welch’s t-test (Table 3). After adjustment for multiple comparisons using the Bonferroni-Holm method, none of the differences reached statistical significance. In GLIM-negative patients, no meaningful differences were observed between the intervention and control groups for any parameter: FFMI (mean difference -0.03; adjusted p = 1.0), BMI (–0.17; adjusted p = 1.0), TBW (0.19; adjusted p = 1.0), FM (0.05; adjusted p = 1.0), and phase angle (0.02; adjusted p = 1.0).

**TABLE 3.** Comparison of body composition changes (3rd–1st measurement) between intervention and control groups, stratified by GLIM status

Parameter	GLIM	Mean difference (intervention – control)	t-value	Diference	p-value	Adjusted p-value	Lower limit	Higher limit
FFMI	0	-0.03	-0.44	270.1	0.662	1.0	-0.18	0.12
FFMI	1	0.30	2.43	104.0	0.017	0.2	0.05	0.54
BMI	0	-0.17	-0.94	295.1	0.350	1.0	-0.54	0.19
BMI	1	0.43	1.44	96.4	0.154	1.0	-0.16	1.02
TBW	0	0.19	0.69	284.9	0.492	1.0	-0.36	0.75
TBW	1	-0.51	-0.88	83.6	0.380	1.0	-1.66	0.64
FM	0	0.05	0.13	309.8	0.894	1.0	-0.72	0.82
FM	1	0.21	0.16	84.2	0.869	1.0	-2.35	2.78
phase angle (°)	0	0.02	0.33	257.8	0.744	1.0	-0.09	0.13
phase angle (°)	1	-0.25	-0.25	67.1	0.806	1.0	-0.04	0.31

1 BMI = body mass index (kg/m^2^); FM = fat mass (%); FFMI = fat-free mass index (kg/m^2^); GLIM = Global Leadership Initiative on Malnutrition; TBW = total body water (%)

In GLIM-positive patients, the intervention group showed a greater increase in FFMI compared to controls (mean difference 0.30; 95% CI 0.05–0.54; unadjusted p = 0.017), but this did not remain significant after Bonferroni-Holm correction (adjusted p = 0.2). No significant differences were observed for BMI (0.43; adjusted p = 1.0), TBW (-0.51; adjusted p = 1.0), FM (0.21; adjusted p = 1.0), or phase angle (-0.25; adjusted p = 1.0).

## Discussion

This study evaluated the effects of a combined nutritional and exercise intervention on body composition in breast cancer patients undergoing treatment, stratified by baseline nutritional status according to GLIM criteria. The main findings were that: (1) the intervention did not produce statistically significant improvements in any BIA-derived body composition parameter over the 12-month follow-up; (2) a trend toward greater FFMI increase was observed in GLIM-positive patients in the intervention group (mean difference 0.30; unadjusted p = 0.017), although this did not survive correction for multiple comparisons; and (3) both GLIM-positive and GLIM-negative patients experienced a decline in phase angle during treatment, regardless of group allocation.

### Interpretation of body composition findings

The absence of statistically significant body composition improvements in the overall cohort, despite the multimodal intervention design, warrants careful interpretation. Several mechanistic and practical factors may explain this finding. First, the catabolic effects of cancer treatment-mediated by pro-inflammatory cytokines and tumor-derived factors – may have overwhelmed the anabolic stimulus provided by nutrition and exercise. Systemic inflammation drives accelerated proteolysis and impairs muscle protein synthesis even in the presence of adequate nutritional intake, a hallmark feature distinguishing cachexia from simple starvation.^6,7^ Second, the exercise component of the intervention (twice-weekly 30-minute group sessions plus weekly yoga) may have been insufficient in intensity and individualization to produce measurable changes in muscle mass. This interpretation is supported by the comparison with Short *et al*., who demonstrated a statistically significant increase in phase angle using a more intensive 12-week program comprising 45 minutes of resistance training, 30 minutes of aerobic exercise, and 15 minutes of stretching, three times per week.^22^ Third, treatment-related side effects likely limited adherence to nutritional targets, reducing effective protein and energy intake below prescribed levels.^9,10^

These findings are consistent with those of van der Werf *et al*., who monitored muscle mass changes using CT imaging in patients with metastatic colorectal cancer over a 12-week chemotherapy period and likewise found no statistically significant differences between intervention and control groups despite individualized dietary counseling.^23^ Similarly, Pérez-Bilbao *et al*. reported heterogeneous effects of combined exercise and diet interventions in breast cancer patients, with outcomes depending on intervention type, timing, and patient characteristics.^24^

### GLIM-stratified analysis

The observation that FFMI increased in GLIMpositive patients in both groups, with a greater increase in the intervention group (mean difference 0.30), while it slightly decreased in GLIM-negative patients, is clinically noteworthy despite not reaching statistical significance after correction for multiple comparisons. This differential response may reflect the fact that GLIM-positive patients, who received individualized nutritional counseling as standard care in both groups, benefited more from targeted individualized nutritional support than GLIM-negative patients who received only group-based lectures.

These findings align with those of Kaegi-Braun *et al*., who confirmed the strong prognostic value of GLIM-defined malnutrition for predicting adverse clinical outcomes and suggested – although not statistically significantly – that nutritional intervention may reduce unfavorable consequences among GLIM-positive patients.^19^ Similarly, Jiang *et al*. demonstrated that nutritional support in geriatric inpatients classified by GLIM criteria yielded greater improvements in BMI, handgrip strength, and calf circumference among patients at risk of malnutrition compared to well-nourished patients, suggesting that individualized nutritional support is more effective in malnourished populations.^25^ The EFFECT trial by Schuetz *et al*. further demonstrated that individualized nutritional support in medical inpatients at nutritional risk significantly improved clinical outcomes, reinforcing the concept that baseline nutritional status modifies intervention response.^26^

Altogether, these findings collectively highlight the importance of routine nutritional screening and diagnosis using GLIM criteria, particularly in vulnerable patient populations, and suggest that individualized rather than group-based nutritional approaches may be necessary to achieve meaningful body composition improvements.

### Phase angle

Phase angle decreased in both groups across GLIM subgroups, with no statistically significant differences between intervention and control groups (adjusted p = 1.0). The decline in phase angle during treatment likely reflects the catabolic effects of chemotherapy on cellular integrity, consistent with the findings of Schmidt *et al*., who investigated the effect of 12-week resistance training on phase angle in 158 breast cancer patients and similarly found no statistically significant intervention effect.^27^

However, other studies have demonstrated that more intensive exercise programs can improve phase angle. Short *et al*. reported a significant increase in phase angle following a 12-week individualized rehabilitation program in breast cancer survivors.^22^ Escriche-Escuder *et al*. similarly demonstrated phase angle improvement after a 12-week exercise program that included twice-weekly resistance training and twice-weekly endurance sessions.^28^ These results suggest that exercise intensity and the inclusion of resistance training are critical determinants of phase angle response, and that the exercise component in the present study may have been insufficient to counteract treatment-induced cellular deterioration.

Phase angle has been established as a reliable prognostic marker in cancer patients, with low values associated with higher mortality and worse clinical outcomes.^16,27^ Pereira *et al*. demonstrated that TBW and the ratio of extracellular to intracellular water independently predict mortality in hospitalized oncology patients.^29^ The clinical relevance of even modest phase angle changes underscores the importance of incorporating this parameter into routine nutritional monitoring.

### BMI and fat mass

Both BMI and fat mass percentage increased in the intervention and control groups, with a greater increase observed among GLIM-positive patients. However, no statistically significant differences were found between subgroups (adjusted p = 1.0). These findings are consistent with those of Ligibel *et al*., who observed nonsignificant reductions in body mass, body fat, and waist circumference in breast cancer patients receiving supervised resistance exercise and aerobic training.^30^

Importantly, BMI and fat mass alone are insufficient indicators of treatment prognosis. Treatment outcomes in oncology patients are more closely related to individual body composition than to BMI.^14^ Simillis *et al*. demonstrated a U-shaped relationship between BMI and survival in colorectal cancer surgery patients, with both very low (18.5 kg/m^2^) and very high (> 35 kg/m^2^) BMI associated with worse outcomes, while overweight patients (BMI 25–30 kg/m^2^) had the best survival.^31^ This further supports the use of body composition parameters such as FFMI and phase angle rather than BMI alone for prognostic assessment.

### Total body water

TBW decreased in both groups and across GLIM subgroups, with a more pronounced decrease in GLIM-positive patients. No statistically significant differences were observed between groups (adjusted p = 1.0). Since TBW is strongly correlated with FFM - in healthy individuals, FFM contains approximately 73% of total body water, changes in TBW may partially reflect changes in lean tissue mass.^32^ The more pronounced TBW decrease in GLIM-positive patients, despite their FFMI increase, may reflect shifts in fluid distribution related to treatment effects or changes in fat mass composition rather than true lean tissue loss.

### Comparison with individualized intervention studies

The contrast between the present study’s null findings and the positive results reported in studies using individualized approaches is instructive. Mlakar-Mastnak *et al*. demonstrated that individualized nutritional intervention by a clinical dietitian in patients at risk of malnutrition in Slovenian primary healthcare produced statistically significant increases in phase angle, FFMI, and BMI after six months.^21,33^ That intervention consisted of four individual consultations over six months with tailored plans based on each patient’s energy, macro-, and micronutrient needs.^21^ The present study’s nutritional program for GLIM-negative patients in the intervention group consisted of group workshops with very low average attendance (31 participants per session), suggesting that group-based approaches may be insufficient to achieve meaningful behavior and nutritional change.

Furthermore, Champ *et al*. reported that resistance exercise has a greater effect on maintaining or increasing muscle mass in cancer patients compared to aerobic exercise alone.^34^ The present study’s exercise component lacked an individualized resistance training component, which may have limited its effectiveness. Incorporating individualized resistance training, as recommended by current evidence, could potentially improve outcomes in future studies.^9,12,13^

### Strengths and limitations

This study has several strengths. The 12-month follow-up period is longer than most previous studies, which typically implemented interventions over 12 weeks.^22,23,27,28^ BIA is a practical, safe, and cost-effective method for body composition assessment compared to DXA, CT, or MRI.^34^ The stratification by GLIM criteria provides novel insights into the interaction between baseline nutritional status and intervention response.

However, several limitations must be acknowledged. First, complete data for all three measurements were not available for all OREH study participants, reducing the sample size from 600 to 481 and potentially introducing selection bias. Second, the proportion of patients with obesity (BMI ≥ 30 kg/m^2^) was approximately 21% in both groups; BIA may not be sufficiently accurate in these patients, potentially underestimating fat mass and overestimating FFM.^35,36^ However, a study comparing BIA and DXA for monitoring FFM changes in cancer patients found 100% sensitivity and 90% specificity for detecting FFM reductions greater than 5%, supporting BIA as a suitable method for detecting clinically meaningful changes.^35^ Third, attendance at intervention workshops was very low (average 25–35 participants per session), and individual attendance tracking was not available, making it impossible to determine the effective intervention dose. Fourth, the significant baseline imbalance in living environment (p = 0.001) represents a potential source of confounding, as urban versus rural residence may affect access to intervention resources, dietary patterns, and physical activity opportunities. Fifth, the study design was retrospective, limiting the ability to establish causality. Sixth, the exercise component lacked individualization and dedicated resistance training, which may have reduced its effectiveness. Finally, chemotherapy regimen heterogeneity introduced unmeasured variability in catabolic stress.

### Clinical implications and future directions

Based on these results and supporting evidence from the literature, several recommendations can be made for future individualized rehabilitation programs for breast cancer patients.^37^ First, routine nutritional screening using NRS 2002 followed by GLIM diagnostic assessment should be standard practice, as baseline nutritional status appears to modify intervention response.^17,18,19^ Second, nutritional interventions should be individualized with concrete goals for energy and protein intake for all patients, not only those diagnosed with malnutrition, as the EFFECT trial demonstrated that individualized nutritional support significantly reduces mortality in at-risk patients.^26^ Third, exercise programs should include resistance training at least two to three times per week, as evidence consistently demonstrates that resistance exercise is more effective than aerobic exercise alone for preserving or increasing muscle mass in cancer patients.^9,12,33^ Fourth, intervention adherence should be tracked individually to enable accurate assessment of effectiveness. Fifth, future studies should consider longer intervention durations, larger sample sizes with adequate statistical power, and multicenter designs to improve generalizability.

## Conclusions

In this study, a combined nutritional and physical activity program did not produce statistically significant improvements in BIA-derived body composition parameters (FFMI, phase angle, fat mass, TBW, and BMI) over 12 months in breast cancer patients undergoing treatment. The intervention, which included group-based nutrition lectures and supervised exercise without individualized resistance training, was insufficient to prevent treatment-related body composition changes. However, a trend toward greater FFMI improvement in GLIM-positive patients, who received individualized nutritional counseling as standard care, suggests that personalized nutritional approaches may be more effective than group-based interventions. Based on these findings and supporting evidence, future programs should incorporate individualized nutrition counseling with specific energy and protein targets for all patients, structured resistance training, and systematic adherence monitoring to optimize body composition outcomes in breast cancer patients.
